# Predictors of Olfactory Impairment among Northern Iranian Population

**DOI:** 10.22038/ijorl.2019.40358.2325

**Published:** 2020-09

**Authors:** Mir Mohammad Jalali, Ali Faghih Habibi, Mehdi Ghorbani Samin

**Affiliations:** 1 *Otorhinolaryngology Research Center, Department of Otolaryngology and Head and Neck Surgery, School of Medicine, Guilan University of Medical Sciences, Rasht, Iran.*; 2 *Guilan * *Legal Medicine Organization, * *Rasht, Iran.*

**Keywords:** Iran, Olfactory disorders, Olfactory perception, Smell, Smell identification test

## Abstract

**Introduction:**

The performance in most smell identification tests is subjected to cultural variations. This study aimed to evaluate age, gender, and smoking-related effects on the test performance in the North of Iran.

**Materials and Methods:**

The olfactory function of 1470 eligible subjects was assessed in this study. Moreover, this study evaluated the influence of age, gender, and education on the test scores.

**Results:**

According to the results, females obtained higher mean test scores, compared to males (18.4 vs. 17.6). In general, the elderly obtained lower scores, and about 30% of the subjects who were ≥65 years of age had severe hyposmia or anosmia. Furthermore, the olfactory impairment frequency in smoker subjects was significantly more than non-smokers (P<0.001). Test scores were generally higher in subjects with higher education levels. Logistic regression analysis revealed that the total number of cigarette doses in smokers and age had a significant association with olfactory dysfunction (P value <0.001 and 0.004, respectively). Cronbach’s α of Iran-SIT was obtained at 0.78 which was more than an acceptable value of 0.7.

**Conclusion:**

The findings of this study revealed that a low score in the Iran-SIT correlated with smoking, older age, low education level, and gender (male).

## Introduction

Olfactory impairment is a public health problem. The researchers estimate the prevalence of this condition between 3.7% and 25% in different populations (1-5). Although many clinical olfactory tests and the majority of the odor identification, detection, discrimination, and memory tests have been described in the literature, only a few have been widely accepted and are available commercially (6). The University of Pennsylvania Smell Identification Test (UPSIT) is a popular test which consists of 40 odors (7). Moreover, it is a useful clinical tool for assessing changes in the olfactory mucosa. In addition, the decreased odor has been associated with alcoholism, early stages of Alzheimer's disease, smoking, sinusitis, cystic fibrosis, Down syndrome, exposure to industrial chemicals, Parkinson's disease, and many others (8,9). One of the main drawbacks of the UPSIT in countries other than the US is that normative values ​​may not be easily transferred from the US to other countries. Other researchers invented several tests, such as the sniffing strength test, the alcohol sniffing test, the 3-element anosmia sniffing test, the sniff-stick test, the Barcelona odor test-24, and cross-cultural smell identification test (10). However, most odor identification tests have a cultural bias, and the same test may not be applied to people of all ages with different backgrounds (11). Therefore, culture-specific adaptations must be applied to most existing tests to allow the use of common normative data (12). Unfortunately, these studies are often confronted with small and unrepresentative sample sizes that do not reflect the underlying structure of the populations to which their results are to be generalized. Since there is no accepted smell test in Iran, Taherkhani et al. (13) have recently developed an olfactory test (Iran smell Identification Test: Iran-SIT) for clinical assessment of olfactory function in Iranian population. In Iran-SIT, items and distractors which appeared to be inappropriate were replaced with items and options believed to be well-known in Iran. The authors assessed the reliability and stability of Iran-SIT in 96 subjects after 5 months. The test-retest analysis revealed that Iran-SIT is a highly reliable and valid test with a Pearson correlation coefficient of 0.93. In this study, Iran-SIT was administered to a large number of residents in Guilan province located in the North of Iran. The selected population reasonably represented a cross-section of the society, and they were selected from those who referred to a local primary health care (PHC) center with different linguistic, cultural, and educational backgrounds. This study aimed to assess the effect of age, gender, and smoking on the test performance in the North of Iran.

## Materials and Methods

This population-based cross-sectional study was conducted between April and August 2017 among residents in urban areas of Guilan province, Iran. Guilan is one of the Northern provinces of Iran with a population of 2,530,692 according to the 2016 census. About 63.3% of this population live in 16 cities. The subjects over the age of 14 years who were living in the region were invited to participate in this study. Moreover, the participants over the age of 64 years should have normal cognitive functioning (Mini-Mental State Examination scores ≥24). The exclusion criteria were: 1) sinonasal disease, 2) a neurological or neuropsychiatric disorder, 3) a history of radiotherapy or chemotherapy, toxic chemical exposure, and head trauma, and 4) consumption of medications affecting olfaction. It should be noted that pregnant women were also excluded from the study. This study was conducted at a local PHC, which is a government system initially designed to provide rural populations and people living in small cities with basic health care in Iran. The study protocol was approved by the review boards of Guilan University of Medical Sciences, Rasht, Iran, (approval id: IR.GUMS. REC.1394. 421), and complied with the principles outlined in the Helsinki Declaration. Informed consent was obtained from all individuals included in the study. The sample size was determined at 1560 subjects considering the 95% confidence level, interval width of 1.2, anticipated population standard deviation 2.00-2.04, gender and age category, and a design effect of 1.5.

**Test Procedures**

A team of 12 trained research specialists administered a 10-item questionnaire detailing basic health, demographic characteristics, and an Iran-SIT to the subjects. The Iran-SIT is the Persian version of the UPSIT. The Iran-SIT, a 24- item odor-microencapsulated odor identification test described in detail elsewhere (13). For a given item, the patient releases an odor by scratching the microencapsulated label with a pencil tip, smells the label, and indicates a name from a set of four odor descriptors.

A response is required for each test item, even if no smell is perceived. The time interval between each sniff was 30 sec. In some cases, the examiner helped administer the test to subjects who could not read or who had impaired eyesight. The test is scored as the number of odors identified correctly. Reference test score was used for olfactory diagnosis, and normosmia was defined as the test score over 18. Moreover, mild microsmia was defined as test scores from 14 to 18, and the test scores from 10 to 13 indicate severe microsmia. Additionally, the scores from 0 to 9 signify anosmia. This test has been shown to be highly reliable (13).

**Statistical Analysis**

Normative data were developed depicting medians, interquartile ranges, and percentiles for the test scores of male or female and each 12 age groups, namely 15-19, 20-24, 25-30, 31-34, 35-40, 41-44, 45-50, 50-54, 55-59, 60-64, 65-69, and ≥70 years. Regarding the original development of the UPSIT (7), the relative effects of age, gender, and education level were also assessed on the test scores. Furthermore, multiple linear regression and logistic regression were performed to evaluate the factors that affected the Iran-SIT test scores in this population. The Iran-SIT was entered as outcome variable, and age, gender, current history of smoking, previous history of smoking, smoking dose (pack-years), and education level were considered as covariates.The education level was considered both continuous (the total number of years of schooling) and categorical (based on quartiles of the variable distribution observed) variable. In this study, subjects at first quartile (Q1) were illiterate, whereas those at the Q4 were bachelors or had higher degrees. A cigarette pack-year was defined as a pack of cigarettes (20 cigarettes) smoked every day for one year. The logistic regression analysis allowed for the calculation of an olfactory dysfunction odds ratio while adjusting for potential confounders, such as age.

The Iran-SIT score was used as a dichotomous outcome (normal or abnormal). Pearson's correlation coefficients were measured, and the reliability of the Iran-SIT was assessed with internal consistency. The internal consistency was evaluated with Cronbach’s α coefficient. The generally acceptable Cronbach’s α was obtained at ≥0.7. All computations were performed using Stata software (version 13.0). A p-value less than 0.05 was considered statistically significant.

## Results

The initial study population was comprised of 1596 volunteers with the age of 14 years and older. In total, 126 subjects were excluded from the study. The reasons for the exclusion were self-reported loss of smell or taste (24 cases), inability to complete the research procedure (36 cases), previous sinus surgery (13 cases), recent upper respiratory infection (13 cases), head trauma (23 cases) and neurological/ neuropsy- chiatric disorder (13 cases). Regarding the sample attrition, eventually, 1470 subjects participated in this study of whom 685 (46.6%) and 785 (53.4%) cases were male and female, respectively. Moreover, about 66.0% and 89.4% of the males and females had never smoked, and approximately, 10% of the participants were illiterate. It should be noted that the illiteracy rate was higher in females (12.5% in females vs. 7.0% in males) and older people (33.5% in subjects ≥ 55 years vs. 2.7% in subjects <55 years). Table 1 summarizes the demographic characteristics of the participants

**Table 1:** Demographic characteristics of the participants

	**Male (n=685)**	**Female (n=785)**	**Total (n=1470)**
Marital status Single Married Widow	160 (23.4) 523 (76.4) 2 (0.2)	178 (22.7) 604 (76.9) 3 (0.4)	338 (23.0) 1127 (76.7) 5 (0.3)
Education level (mean±SD)	10.8(4.8)	10.9 (5.7)	10.9(5.3)
Smoking (n %) Never Previously Currently	452 (66.0) 48 (7.0) 185 (27.0)	702 (89.4) 68 (8.7) 15 (1.9)	1154 (78.5) 116 (7.9) 200 (13.6)
Pack year (mean±SD)	12.1 (2.8)	10.5(2.5)	11.7(2.8)
Mini-Mental State Exam score (mean±SD)	27.7 (1.7)	28.0(1.7)	27.8(1.7)

**Fig 1:**
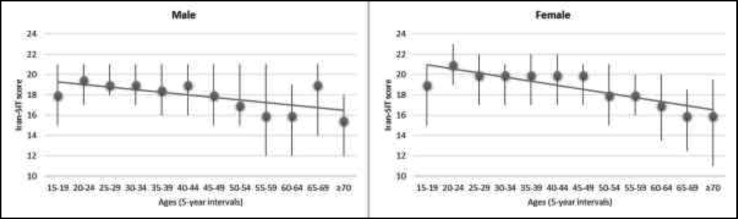
Mean and 95% confidence interval of the Iran-SIT score as a function of age

As can be seen in Table 2 and Figure 1, females obtained higher scores than males (mean female score: 18.6 with 95% confidence interval [CI] 18.3 to 18.8, mean male score: 17.5 with 95% CI 17.2 to 17.9). Regardless of the smoking status, the results for males and females falling below the Iran-SIT normosmic category showed a significant difference between males and females regarding the olfactory dysfunction (male: 55.1% vs female: 49.3%; Chi-square=5.07, P=0.02).

In general, the older people obtained lower scores, and 31.8% (95% CI 25.4%-39.0%) and 26.5% (95% CI 20.4%-33.6%) of the males and females who were ≥ 55 years of age, respectively, had demonstrable severe microsmia or anosmia. This value was about three times higher than that in younger subjects (12.8% in males and 8.6% in females). 

**Fig 2:**
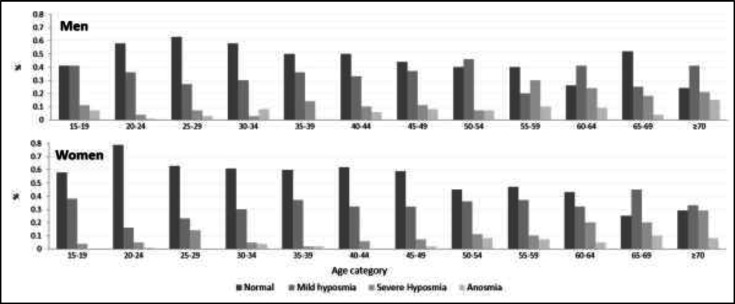
Clinical classification of olfactory deficit in different age groups.

Furthermore, the results revealed that about 30% of the subjects who were ≥65 years of age had severe hyposmia or anosmia (Fig. 2). 

**Table 2:** Scores of the Iranian Smell Identification Test in all groups (total score=24)*

	Male (n=685)		Female (n=785)		Total (n=1470)*		
	Mean	SD	Mean	SD	Mean	SD	% (95% CI) subjects ≥ severe hyposmia
**15-19**	17.5	4.1	19.0	3.0	18.2	3.7	11.7 (5.3-24.0)
**20-24**	19.0	3.1	20.4	3.0	19.9	3.1	5.3 (3.0-9.4)
**25-29**	19.0	3.6	19.1	3.8	19.0	3.7	12.0 (7.7- 18.3)
**30-34**	18.1	4.4	18.8	3.8	18.4	4.1	9.9 (6.2-15.3)
**35-39**	18.2	3.5	19.4	3.1	18.9	3.3	8.3 (4.5-14.8)
**40-44**	17.6	3.9	19.3	3.3	18.6	3.6	9.8 (5.8-16.3)
**45-49**	17.1	4.4	18.8	3.3	18.1	3.9	13.6 (9.2-19.7)
**50-54**	17.3	4.0	17.3	4.3	17.3	4.2	16.5 (10.9-24.3)
**55-59**	16.0	5.2	17.5	4.1	16.7	4.7	30.0 (20.4-41.7)
**60-64**	15.7	4.3	16.8	4.1	16.4	4.2	28.5 (21.3-36.8)
**65-69**	17.3	4.2	15.6	4.1	16.4	4.2	25.3 (17.4-35.2)
**≥70**	15.2	4.5	15.4	4.5	15.3	4.5	36.2 (24.8-49.4)
**Total**	17.5	4.1	18.6	3.9	18.1	4.0	15.0 (13.2-16.9)

* All values are adjusted as the frequency of age category and smoking status in the North of Iran SD: Standard Deviation

Moreover, olfactory impairment frequency (≥severe microsmia) in smoker subjects was significantly more than that in non-smokers (22.5% vs. 13.2%, P<0.001). Although the subjects with the previous history of smoking had a better score than current smokers, olfactory impairment frequency was more than that observed in nonsmokers (19.8% vs. 13.2%, P<0.001) (Table 3). The results of the logistic regression analysis revealed that the total number of cigarette doses in smokers (either previous or current) associated significantly with the category of Iran-SIT (β=-0.25, P<0.001). Females are nearly half less likely to have an olfactory dysfunction than males (adjusted odds ratio 0.51, 95% CI 0.28-0.91, P=0.02). Additionally, there was a significant reverse correlation between total scores of Iran-SIT and the total number of cigarette dose (r=-0.36, P<0.001). 

**Table 3:** Clinical classification of olfactory deficit according to smoking status in males and females*

		Smokers (n _male_=185, n _female_=15)	No Smokers (n _male_= 500, n _female_=770)	Total (n _male_= 685, n _female_=780)
**Normal**	Male Female	83 (44.9) 8 (53.3)	224 (44.8) 390 (50.6)	307 (44.8) 398 (50.7)
**Mild Microsmia**	Male Female	59 (31.9) 2 (13.3)	191 (38.2) 273 (35.5)	250 (36.5) 275 (35.0)
**Severe Microsmia**	Male Female	32 (17.3) 4 (26.7)	49 (9.8) 78 (10.1)	81 (11.8) 82 (10.4)
**Anosmia**	Male Female	11 (5.9) 1 (6.7)	36 (7.2) 29 (3.8)	47 (6.9) 30 (3.8)
**Total**	Male Female	185 (100) 15 (100)	500 (100) 770 (100)	

* Frequency was shown as number (percentage of total smokers and no-smokers) for males and females

**Fig 3:**
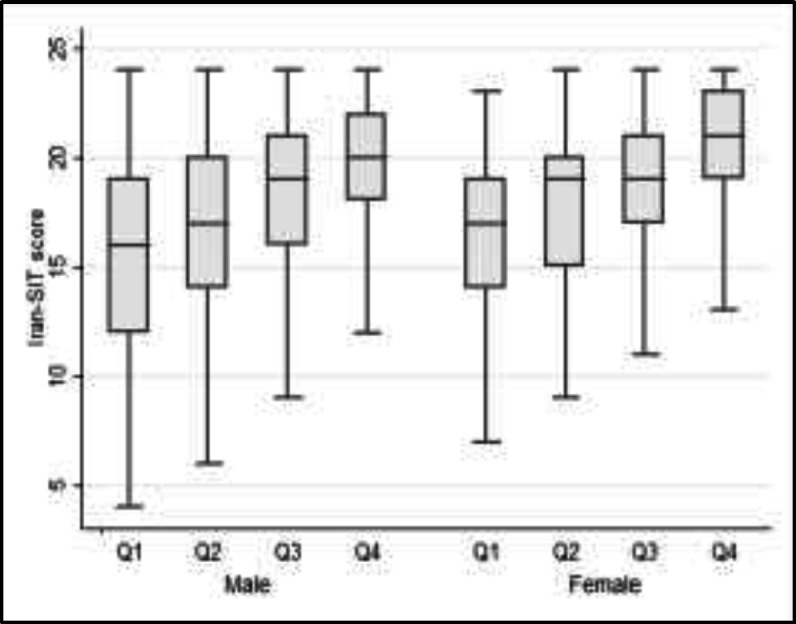
Comparison of Iran-SIT scores among male and female subjects with different levels of education (Q1=lowest level of education, Q4= highest level of education).

The results showed that males and females at Q1 of education level obtained the mean scores of 15.7 (±4.8) and 16.2 (±3.9), respectively. However, those at the Q4 achieved the mean scores of 18.9 (±3.6) and 20.2 (±3.2), respectively. Therefore, the Iran-SIT scores seemed to be generally higher in subjects with a higher education level (Fig. 3).

The multiple linear regression using the Iran-SIT as the outcome variable showed that age (β coefficient 0.06, 95% CI 0.02 to 0.10, P=0.004), education level (β coefficient 0.15, 95% CI 0.05 to 0.25, P=0.002), smoking (β coefficient -2.54, 95% CI: -3.8 to -1.3, P<0.001), and total number of cigarette dose (β coefficient -0.81, 95% CI -1.04 to -0.58, P<0.001) were independent predictors of the Iran-SIT scores, except for gender (P=0.66). Although the olfactory function was depressed in males, logistic regression analysis failed to find a significant association between gender and olfactory dysfunction (P=0.85). Some of the variables in the first multiple linear regression were interrelated, and age correlated negatively with education level (r=-0.56, P<0.001). However, the Iran-SIT score was positively associated with education level (r=0.34, P<0.001). In addition, the t-test revealed no difference between males and females in terms of education level (P=0.72), and the Chi-square test showed a significant difference between genders regarding the proportion of subjects with a current or previous history of smoking (P<0.001). 

Reliability analysis of the data collected in the present study showed good Cronbach’s alpha values (0.78). However, results for individual smells showed that less than 70% of the female participants correctly identified five out of 24 odors, including saffron (51.9%), onion (66.5%), peanut (49.8%), cinnamon (59.9%), and coconut (67.8%). In addition, the rate of correct identification was higher than 90% in items 2 and 16 (i.e., bubble gum and garlic, respectively).

## Discussion

Olfaction is most commonly measured in the clinic using odor identification tests (14). Most of these tests use odors in the form of microencapsulated odor strips, pen-like devices, squeeze bottles, or sniff bottles (6).

The patient is asked to identify the odor, usually by choosing an answer from a shortlist of written alternatives. Among clinical olfactory tests, the UPSIT is the most widely used smell test in the world. However, it is influenced by cultural factors, which have led to the development of Iran-SIT. The present study, which is the largest clinical study of olfaction, employed a highly reliable and standardized Iran-SIT in a representative sample of the Iranian population to date. This study aimed to evaluate the effects of age, gender, smoking, and other socioeconomic factors on olfaction. 

Cronbach’s α of the Iran-SIT was obtained at 0.78 in our population which was more than an acceptable value of 0.7. Since the test length relates to reliability, lower value compared to 92% reliability of UPSIT could be anticipated (15). In our study of healthy Iranian subjects, five odors were correctly identified by less than two-thirds of males and females. It seems that familiarity with the test items due to different cultures is a significant contributor. The use of distractors with odors dissimilar to that of the correct choice undoubtedly widens the differences between the performance of the microcosmic and anosmic subjects. 

Moreover, it was revealed that the elderly obtained lower scores, and about one-third of the subjects who were over the age of 64 years experienced severe hyposmia or anosmia. The same prevalence rate is observed in some studies (1,16). However, due to the design of the current study, the prevalence of age-related olfactory dysfunction was probably underestimated (7,17). 

Doty and Kamath reviewed different factors that likely contribute to such changes as age-related alterations within the nose, olfactory epithelium, bulb, and higher brain structures (18).Consistent with the findings of the previous smell identification studies (19-23), it was found that females obtained higher Iran-SIT scores, compared to males (18.4 vs 17.6, respectively). The prevalence of severe hyposmia or anosmia was more pronounced in males than that in females (Table 2). The basis for gender differences in odor identification is not known; however, it appears that complex relationships exist between the functional properties of the olfactory system and a range of interacting neuroendocrine factors during early brain development and at later stages of life. Schlaepfer et al. (24) assessed gray matter volumes in several cortical regions using magnetic resonance imaging. They found that women had 23.2% (dorsolateral prefrontal cortex) and 12.8% (superior temporal gyrus) gray matter percentages higher than men in a language-related cortical region. Therefore, the higher-order cortical difference between genders could reflect in cognitive functional difference. In general, olfactory measurements in which semantic memory plays a role show greater gender differences, compared to those in which semantic memory plays no role (20,25). In our study, no significant association was observed between gender and olfactory dysfunction using multiple linear regression and logistic regression analysis. Recently, Sorokowski et al. (26) performed a meta-analysis about gender differences. This meta-analysis showed that olfactory threshold tests were the most appropriate to assess gender differences in olfaction and less prone to the influence of verbal components. 

Another explanation for this observation is that the initial effects of gender on odor function were mitigated by other factors, including the education level. Compared to males, females had a lower education level, and about 12% of the females were illiterate. Therefore, the choices were presented orally instead of written form, which could lead to a low-performance level. It is well known that education influences cognitive abilities, such as executive functioning and semantic memory, thereby positively influencing on applying testing strategies in performing the olfactory tests (27,28).

 In our study, the results of the multiple linear regression showed that the education level predicted the Iran-SIT score significantly (P=0.002). It is worth mentioning that oral contraceptive use and menopausal status were not evaluated in female subjects. Therefore, it is impossible to elucidate the true effects of gender on odor identification.

There is controversy about the effect of smoking on the olfactory function. Contrary to early studies, recent researches have shown the effect of smoking on olfactory sensitivity (3,29,30). The cigarette dose in pack-years was calculated in this study. Furthermore, subjects who were smokers in the past were also taken into account. Our analysis showed that smoking predicts the Iran-SIT score, and this association is dose-related. The dose-related decrease in the olfactory function of smokers was supported by the literature (29,31). A significant number of current and previous smokers were observed to be severe microsmic or anosmic. This finding is in agreement with the results of previous studies (29,31). Although Frey et al. (31) reported only mild or moderate and not severe olfactory loss from smoking, a recent study conducted by Katotomichelakis et al. (29) showed a 5-fold higher independent risk for the dysfunction of identification ability among smokers, compared to non-smokers. In contrast with our findings, Bramerson et al. (1) evaluated olfactory dysfunction in an adult Swedish population and failed to find an increased risk for current smokers or the number of pack-years. The authors proposed that smoking affected the olfaction of certain substances more than others.

The biological basis for the decreased ability to smell associated with smoking is not known. The influence of the chemicals in cigarette smoke on the olfactory receptor cells might be short- or long-term within the olfactory mucosa. Short-term effects could be caused by the changes in the consistency or nature of the mucus overlying the receptors, and possibly, adaptation or habituation of the receptor system (32,33). Several potential mechanisms have been proposed for the recovery of odor identification in previous smokers, including the reversibility of metaplastic changes in response to insults from tobacco smoke and resolution of acute or chronic inflammation (29). In addition to the direct effects of tobacco smoke on the peripheral olfactory system in the nose, neurotoxic effects of tobacco smoke have been observed on cognition (34). It is well known that odor identification tasks have a cognitive component and cessation of smoking may result in improved olfaction (35). Long-term effects could be caused by the adverse influence of chemicals in cigarette smoke on the olfactory receptor cells within the olfactory mucosa. It is well known that animals exposed to brief exposures to cigarette smoke exhibited anatomic changes of the olfactory mucosa (36-38). Increased activity of neuronal apoptosis in olfactory epithelium has been demonstrated in animals exposed to tobacco smoke (38).

Regarding the limitations of this study, it should be noted that the study population was grouped into non-smokers, as well as previous and current smokers. Moreover, the smoking dose was measured as the number in pack-year. However, more smoking data, such as cigarettes per day and time since quitting smoking are potentially important factors. Furthermore, the association between environmental tobacco smoke and olfaction was not evaluated in this study. Another limitation is given by the cross-sectional study design which does not allow to establish a time sequence between the risk factors (i.e., smoking) and smell impairment. Finally, the odor identification task of subjects was only measured in this study, and further studies are required to assess other domains of olfaction, such as odor threshold and discrimination. 

## Conclusion

The present study provides normative data for assessing olfactory function in the Iranian population. The sample in this study represented a range of ages and varying degrees of education. The results showed an association between diminished olfactory sensitivity and cigarette smoking along with a direct negative correlation between olfactory sensitivity and the number of smoked cigarettes. 
